# A comparison of leg length and femoral offset discrepancies in hip resurfacing, large head metal-on- metal and conventional total hip replacement: a case series

**DOI:** 10.1186/1749-799X-6-65

**Published:** 2011-12-29

**Authors:** Katie A Herman, Alan J Highcock, John D Moorehead, Simon J Scott

**Affiliations:** 1 University of Liverpool Liverpool L69 3BX, UK; 2 Trauma and Orthopaedic Department University Hospital Aintree Longmoor Lane Liverpool, L9 7AL, UK

**Keywords:** Hip resurfacing, total hip replacement, leg length, femoral offset

## Abstract

**Background:**

A discrepancy in leg length and femoral offset restoration is the leading cause of patient dissatisfaction in hip replacement surgery and has profound implications on patient quality of life. The aim of this study is to compare biomechanical hip reconstruction in hip resurfacing, large-diameter femoral head hip arthroplasty and conventional total hip replacement.

**Method:**

Sixty patient's post-operative radiographs were reviewed; 20 patients had a hip resurfacing (HR), 20 patients had a Large Head Metal-on-metal (LHM) hip replacement and 20 patients had a conventional small head Total Hip Replacement (THR). The leg length and femoral offset of the operated and unoperated hips were measured and compared.

**Results:**

Hip resurfacing accurately restored hip biomechanics with no statistical difference in leg length (*P *= 0.07) or femoral offset (*P *= 0.95) between the operated and non-operative hips. Overall HR was superior for reducing femoral offset discrepancies where it had the smallest bilateral difference (-0.2%, *P *= 0.9). The traditional total hip replacement was least effective at restoring the hip anatomy.

**Conclusion:**

The use of a larger-diameter femoral head in hip resurfacing does not fully account for the superior biomechanical restoration, as LHM did not restore femoral offset as accurately. We conclude that restoration of normal hip biomechanics is best achieved with hip resurfacing.

## Background

Each year around 72,000 hip replacements are performed across the UK [1]. This number is steadily rising and is predicted to increase by 40% over the next 30 years due to the ageing population [2]. The National Institute for Clinical Excellence (NICE) recommends hip resurfacing in patients under 65 years old with severe hip disease who may outlive the standard small head THR [3]. However, there is a debate over which type of hip replacement provides the best outcome with regards to restoration of leg length and femoral offset.

One of the main challenges of hip replacement is to restore leg length and provide optimal femoral offset. Even with the new techniques and technology available to aid this, it still proves to be technically challenging. A difference in operated and unoperated leg length creates tension in the soft tissue structures and muscles around the operated hip. This causes the pelvis to tilt, creating a sensation that one leg is longer [4]. A leg length discrepancy can lead to low back pain, discomfort, instability, abnormal gait, nerve palsies and patient dissatisfaction [5]. A difference in the femoral offset postoperatively is often the result of the larger neck-shaft angle of the prosthesis than the patient's own anatomy [6]. The femur moves closer to the pelvis and reduces both the range of movement [6] and the tension on surrounding soft tissues. A low femoral offset can lead to wearing of the acetabular cup which is the primary cause of aseptic loosening [6], abnormal gait, joint instability [7] and dislocation [8].

A discrepancy in such restoration is the leading cause of patient dissatisfaction [6] and has profound implications on patient quality of life. Therefore it is important that further research is undertaken in this area.

In our study we followed up patients who had undergone hip arthroplasty. The aim was to find out which type of hip replacement best reduced leg length and femoral offset discrepancy postoperatively.

## Methods

**Figure 1:**
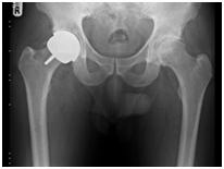
**Radiograph showing hip resurfacing**.

**Figure 2:**
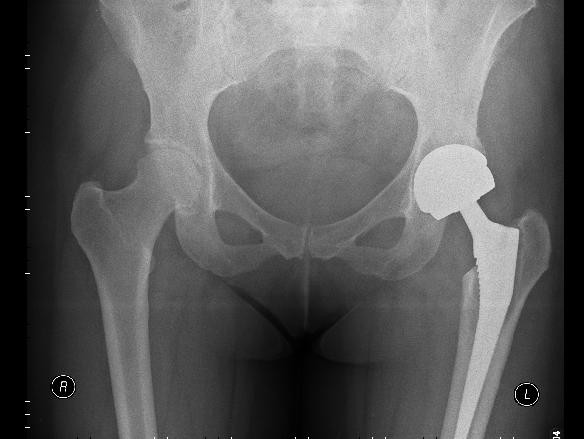
**Radiograph showing a LHM replacement**.

**Figure 3:**
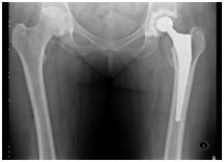
**Radiograph showing a small head THR**.

We selected a total of 60 patients from the surgical register of hip replacements;

• 20 patients had a large MoM head-Articular Surface Replacement (ASR) hip resurfacing (Figure 1)

• 20 had a LHM-corail with ASR Extra Large (XL) (54 mm) head (Figure 2)

• 20 had a poly-metal THR-corail with Charnley (28 mm head) cemented cup (Figure 3)

These operations were performed by one surgeon, from January 2007 to December 2008. A posterior approach to the hip replacement was used for all patients. Prior to each surgical case the patients radiograph was templated using *traumaCAD *with the aim of accurately restoring both leg length and femoral offset with respect to the contralateral hip.

Inclusion criteria included patients with primary hip procedures, one unoperated and one operated hip and patients with any of the three types of hip replacements. Exclusion criteria included patients with an abnormal unoperated hip e.g. decreased joint space, indefinable anatomical landmarks e.g. acetabular teardrop, or previous femoral fractures.

The PACS-based (Picture Archiving and Communication Systems) x-ray computer program was used which enabled straight lines to be drawn on the radiographs, with their corresponding lengths being recorded in millimeters. The patients' most recent anteroposterior pelvic radiograph (taken at around 6 week postoperatively) was used. The unoperated hip provided control data for comparison with the operated hip. Both the leg length and femoral offset were measured on each hip; operated and unoperated.

**Figure 4:**
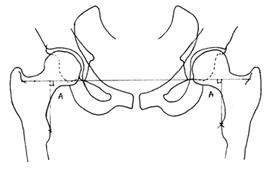
**A diagram demonstrating the method of measuring leg length**. A. Leg length measurement. Sources of both diagram 4 and 5, used with permission: Barrett MP, Griffiths P, Couch, M. Modular vs. Non-Modular: Which is More Effective in Restoring Femoral Offset and Leg Length? 2007; 1-33.

Leg length was measured by drawing a straight line across the inferior point of each acetabular teardrop. Two perpendicular lines are drawn from the most medial part of each lesser trochanter superiorly to meet the first line drawn (see Figure 4). This is the standard method of measuring leg length as described by Ranawat et al [9].

**Figure 5:**
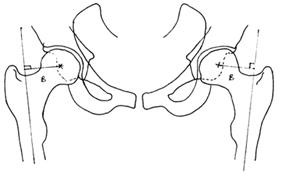
**A diagram demonstrating the method of measuring femoral offset**. B. Femoral offset measurement. Sources of both diagram 4 and 5, used with permission: Barrett MP, Griffiths P, Couch, M. Modular vs. Non-Modular: Which is More Effective in Restoring Femoral Offset and Leg Length? 2007; 1-33.

Femoral offset was calculated by measuring the perpendicular distance from the centre point of the femoral head to a line bisecting the length of the femur [8] (see Figure 5). Moses' concentric circles method was used to find the central of rotation of the femoral head [10].

A leg length difference of up to ± 10 mm and femoral offset of up to ± 4.62 mm were considered acceptable. Woolson at al [5] and Krishnan et al [11] showed in their studies that a discrepancy of more than such measurements has been shown to significantly increase the risk of long-term complications.

Each measurement was made by one investigator on two separate occasions which gave an indication of intra-observer repeatability. A second observer then re-measured all the radiographs to provide an indication of inter-observer reproducibility. The Pearson correlation coefficient was used to assess intra-observer repeatability and inter-observer reproducibility. The Munro classification system was used to interpret the correlation co- efficient scores [12].

The two sets of measurements from observer 1 were averaged to give mean measurements of leg length and offset for each of the three arthroplasty groups. The measurements were analysed using the Student's paired t-test to see if the bi-lateral comparisons in each group were statistically significant.

## Results

All three types of implant appeared to adequately restore pre-operative leg length (Table 1).

**Table 1:** The mean leg lengths, their postoperative discrepancy, % acceptable and statistical significance

	Leg length (mm)				
	
	Operated	Contralateral	Average diff	< 10 mm diff	P value
**Hip Resurfacing**	52.13	49.35	-2.78	95%	0.07
**Large-head metal on metal**	54.95	53.03	-1.92	80%	0.45
**Total hip replacement**	53.24	49.82	-3.42	80%	0.06

**Figure 6:**
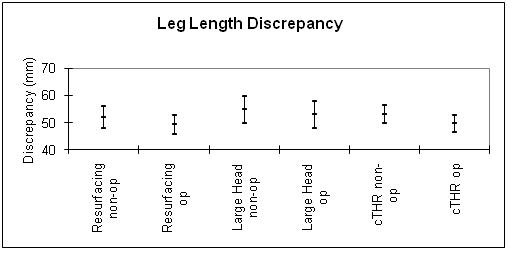
**A graph to show the mean leg length discrepancies using hip resurfacing, LHM and small head THR techniques**.

Figure 6 shows the post-operative leg length discrepancy with 95% confidence interval.

Only the hip resurfacing restored the pre-operative femoral offset (Table 2).

**Table 2:** The mean femoral offsets, their postoperative discrepancy, % acceptable and statistical significance

	Femoral offset (mm)				
	
	Operated	Contralateral	Average diff	< 4.62 mm diff	P value
**Hip Resurfacing**	50.71	50.63	-0.08	50%	0.9
**Large-head metal on metal**	51	56.56	+5.56	35%	0.0002
**Total hip replacement**	47.61	56.23	+8.62	30%	0.0003

**Figure 7:**
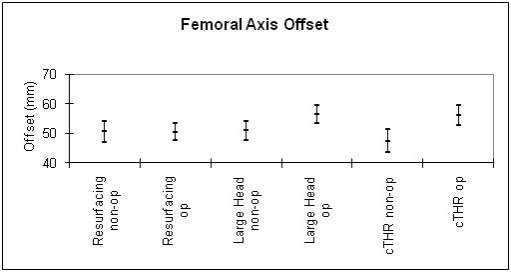
**A graph to show the mean femoral offset discrepancies using hip resurfacing, LHM and small head THR techniques**.

Figure 7 shoes the post-operative femoral offset discrepancy with 95% confidence interval.

In the hip resurfacing group leg length was restored to < 10 mm difference in 95% cases, and femoral offset was restored to < 4.62 mm difference in 50% of cases. Additionally, there was no statistically significant difference observed in either the leg length (*p *= 0.07) or femoral offset (*p *= 0.95) between the operated and non-operative hips.

With LHM hip replacements, leg lengths was restored to within < 10 mm difference in 80% patients and there was no statistically significant difference in leg length in this group. However, there was a statistically significant increase (*P *= < 0.0002) in femoral offset and only 35% patients had their femoral offset restored to within < 4.62 mm. An average of 5.56 mm increase in femoral offset was seen postoperatively.

The conventional small head THR restored 80% patients leg lengths to < 10 mm difference and the results showed no statistically significant difference. However, these prostheses were the least effective in restoring the femoral offset. The difference in femoral offset was statistically significant (*P *= < 0.0003) with an average increase in postoperative femoral offsets of 8.62 mm. Only 30% patients postoperative femoral offsets were restored to < 4.62 mm.

The large head metal-on-metal hip replacement showed the smallest reduction in leg length of, on average, 1.92 mm compared to the other types of hip replacement. The ranges of results from the other types of hip replacement were similar. Overall all of these hip replacements showed a non- significant difference in leg length between the unoperated and operated leg.

Overall hip resurfacing provided the best results compared to other hip replacement techniques examined in this study, in terms of meeting the set standards of ≤ 10 mm difference in leg length (19/20 patients) and ≤ 4.62 mm difference in femoral offset (10/20 patients). Hip resurfacing had the highest percentage of patients meeting these standards and the lowest average change in leg length and femoral offset. It is therefore superior in restoring hip biomechanics than LHM or conventional small head THRs.

All results for the three arthroplasty groups were pooled into leg length and offset data, for each of the 3 repeated measurements. Correlations were then performed to quantify the intra and inter observer errors. As shown in table 3, there was a very high intra-observer repeatability and high inter-observer reproducibility. This suggests results were therefore reliable.

**Table 3:** The average intra-observer error and inter-observer error for each measurement and the overall mean

	MOM	LHM	THR	Mean
**Intra-observer error**				
Leg length	0.95	0.95	0.97	0.95
Femoral offset	0.82	0.87	0.93	0.88
**Inter-observer error**				
Leg length	0.83	0.93	0.83	0.85
Femoral offset	0.78	0.87	0.94	0.88

## Discussion

The LHM hip replacement tended to restore leg length and hip resurfacing restored femoral offset the most accurately (Table 1). The newer hip resurfacing showed the smallest change in femoral offset with an average difference of -0.08 mm (a non-significant difference). This is contrary to previous studies, where femoral offset has consistently been found to be significantly reduced in hip resurfacing, with variable effects on leg length. This may relate to a tendency to place the femoral head component into a valgus alignment (thereby reducing femoral offset and increasing leg length), to avoid varus alignment, which itself, is associated with increased risk of femoral neck fracture. In our study, the aim was to accurately align the femoral component, matching the patient's own anatomy.

The other two hip replacements, large head metal-on-metal and small head THRs showed a significant difference between the operated and unoperated femoral offsets (Table 2). This indicates that the concept of hip resurfacing is superior in restoring hip biomechanics. Additionally, hip resurfacing provides better stability due to the large-diameter femoral head. It also demands less bone resection from the femoral head, with preservation of the femoral neck when compared to the other two techniques described in this paper, it therefore is less likely to alter the femoral offset [13].

Altogether 19/20 patients with hip resurfacing and 4/20 patients with large head metal-on-metal and small head THR replacement met the set standard for leg length restoration. This shows hip resurfacing was superior at reproducing leg length. The one patient who did not meet the set standard after hip resurfacing had a large difference in leg length of -19.19 mm. This is an anomaly which affected the overall average result for this group. If this measurement was excluded from the study then hip resurfacing would show the smallest reduction in leg length rather than the large head metal hip replacement.

Girard et al [14] performed the only prospective randomised trial on this subject. They compared hip resurfacing and small head THR in two homogenous groups of 120 patients. Similarly to our study they also showed hip resurfacing produced less discrepancy in leg length and femoral offset than small head THR. They concluded that hip resurfacing was superior because the anatomy of the hip is less distorted during the surgery and the large metal head provides hip stability. Overall, the study by Girard et al [14] favours hip resurfacing to reduce leg length and femoral offset discrepancy.

Research by Silva et al [15] looked at the leg length and femoral offset discrepancies in pre and postoperative radiographs of 90 patients who underwent small head THR and hip resurfacing. They found that the leg length and femoral offset discrepancy was higher in hip resurfacing. Silva et al [15] concluded that small head THR was more suitable than hip resurfacing for patients who have a either a preoperative leg length discrepancy of more than 10 mm or a low femoral offset.

Loughead et al [16] also reviewed postoperative radiographs of 54 patients who underwent small head THR and hip resurfacing. They reported an increase in leg length with hip resurfacing, concluding that resurfacing did not produce more accurate restoration of hip biomechanics, and that the advantage of hip resurfacing was likely related to the larger femoral head. This theory has not been supported by our findings.

The limitations of this study include the stringent inclusion/exclusion criteria which eliminated many patients. This accounted for the small sample size and limited the internal validity. There is some selection bias as the participants were chosen from one surgeon and one institution. This limits the external validity of the study. Furthermore, the study's methodology provided level IV evidence and therefore the results should be interpreted carefully.

When deciding which surgical hip replacement technique is superior it is also necessary to evaluate clinical improvement, survivorship, longevity and peri-operative factors including surgical time, hospital stay, complications, total blood loss and costs (£5515 for hip resurfacing, £4195 for hip replacements [17]). Hip resurfacing carries an increased risk of femoral neck fractures, aseptic loosening and metal wear [18]. However, hip resurfacing reduces the risk of postoperative hip dislocation due to its larger femoral head and allows easier revision surgery to a small head THR due its increased bone stock [19]. A randomised controlled trial by Loughead et al [20] showed an 82% clinical improvement and 7% perioperative complications in 35 patients undergoing hip resurfacing compared to 79% and 13% respectively in 33 patients with a small head THR.

## Conclusion

This study provides further evidence that the more contemporary hip resurfacing is superior for restoring leg length and reducing femoral offset discrepancies. It is likely that hip resurfacing further preserves the anatomy of the hip, affords greater stability due to the large-diameter femoral head and improves soft tissue tensions around the hip joint. This may explain the observed increased patient satisfaction with resurfacing arthroplasty.

The lack of studies comparing large head hip replacements to other types indicate that further research is needed. With the increasing number of patients undergoing hip replacements each year there is a need to identify the best yet cost-effective type of hip replacement and indications for its use.

Please note: Since this study was undertaken in 2009 the Johnson & Johnson DePuy MOM hip resurfacing (ASR hip resurfacing system) and LHM (ASR XL head acetabular system) hip implants have been recalled. The metal components have been found to be wearing away and releasing cobalt and chromium ions into the bloodstream of some patients. This has been linked to pain, inflammation, bone and soft tissue damage [21]. Currently these groups of patients are being followed up closely with clinical review, cobalt-chromium ion level checks and Magnetic Resonance Imaging (MRI) scans. The results will be reported and made available as a follow up study.

## Consent

According to the National Research Ethics Committee algorithm (August 2011) this work is classified as a clinical audit. Hence, ethical approval was not needed. In addition, patients were not identifiable and therefore patient consent was not obtained.

## Competing interests

The authors declare that they have no competing interests.

## Authors' contributions

KH collected study data and wrote up the paper. AH collected a set of data for comparison. JM helped with the statistical analysis. SS conceived the study and performed the operations. All authors read and approved the final manuscript.
